# Characterization of Taxonomic and Functional Dynamics Associated with Harmful Algal Bloom Formation in Recreational Water Ecosystems

**DOI:** 10.3390/toxins16060263

**Published:** 2024-06-07

**Authors:** Faizan Saleem, Rachelle Atrache, Jennifer L. Jiang, Kevin L. Tran, Enze Li, Athanasios Paschos, Thomas A. Edge, Herb E. Schellhorn

**Affiliations:** Department of Biology, McMaster University, 1280 Main St W., Hamilton, ON L8S 4L8, Canada; saleef4@mcmaster.ca (F.S.); atracher@mcmaster.ca (R.A.); jiangl55@mcmaster.ca (J.L.J.); trank38@mcmaster.ca (K.L.T.); lie40@mcmaster.ca (E.L.); paschos@mcmaster.ca (A.P.); edget2@mcmaster.ca (T.A.E.)

**Keywords:** harmful algal blooms, metagenomics, microcystin, next-generation sequencing, microbiology

## Abstract

Harmful algal bloom (HAB) formation leads to the eutrophication of water ecosystems and may render recreational lakes unsuitable for human use. We evaluated the applicability and comparison of metabarcoding, metagenomics, qPCR, and ELISA-based methods for cyanobacteria/cyanotoxin detection in bloom and non-bloom sites for the Great Lakes region. DNA sequencing-based methods robustly identified differences between bloom and non-bloom samples (e.g., the relative prominence of *Anabaena* and *Planktothrix*). Shotgun sequencing strategies also identified the enrichment of metabolic genes typical of cyanobacteria in bloom samples, though toxin genes were not detected, suggesting deeper sequencing or PCR methods may be needed to detect low-abundance toxin genes. PCR and ELISA indicated microcystin levels and microcystin gene copies were significantly more abundant in bloom sites. However, not all bloom samples were positive for microcystin, possibly due to bloom development by non-toxin-producing species. Additionally, microcystin levels were significantly correlated (positively) with microcystin gene copy number but not with total cyanobacterial 16S gene copies. In summary, next-generation sequencing-based methods can identify specific taxonomic and functional targets, which can be used for absolute quantification methods (qPCR and ELISA) to augment conventional water monitoring strategies.

## 1. Introduction

Harmful algal bloom (HAB) formation is a natural event caused by the mass increase in phytoplankton proliferation in water ecosystems and is mainly due to Cyanobacteria, Diatoms and Dinoflagellates [1]. Among the major constituents of algal blooms, Cyanobacteria (blue-green algae) are of particular concern for the sustainability of freshwater ecosystems. Under favorable environmental conditions, Cyanobacteria can mass proliferate, thus leading to the deterioration of recreational water quality [2,3]. Several cyanobacterial species can produce cyanotoxins, leading to HABs, rendering the water ecosystems toxigenic and unusable for recreational purposes [4]. Environmental monitoring agencies monitor recreational waters for the formation/persistence of HABs and, depending on the detection methods/water quality thresholds, post warnings at those water bodies used for recreational purposes [5]. Closure of water bodies can last for months and ultimately result in recreational and economic losses [6]. However, bloom formation by itself may not necessarily be indicative of toxigenic Cyanobacteria [7], leading to unnecessary postings. Therefore, it is imperative to have robust HAB detection methods to augment existing water monitoring strategies.

Conventional detection methods are based on microscopy for cyanobacterial cell identification and cell counting [8], polymerase chain reaction for cyanobacterial/cyanotoxin gene copies detection/quantification [9], liquid chromatography/mass spectrometry (LC/MS) and enzyme-linked immunosorbent/chromatography assays for direct assessment of cyanotoxins [10]. Microcystin, the most commonly found cyanotoxin worldwide [11], is a primary focus for detection and monitoring. The United States Environmental Protection Agency (USEPA) has developed a protocol for the determination of total microcystin/nodularins (MCs/NODs) in ambient waters using ADDA-ELISA [12], which targets a conserved part of microcystin. Additionally, the microcystin synthetase (*mcyE*) assay for qPCR can detect all microcystin congeners and the nodularin synthetase gene clusters [13]. In the environment, microcystins have more than 279 variants [14], encoded by the *mcy* gene cluster. PCR-based approaches can be useful in detecting/quantifying cyanotoxin genes and transcripts [15]. There is a strong positive correlation between microcystin gene transcripts and microcystin levels, suggesting PCR-based indicators as potential diagnostic measures [16]. Specifically, a positive correlation between PCR-based measures and microcystin levels has been observed in environmental waters from the US [16,17] and China [18]. However, the relationship between toxins and environmental factors, including internal loading [19] and temperature [20], is well established and has been validated for cyanotoxin prediction models for water ecosystems [19]. Specifically, internal nutrient loading and temperature can be primary drivers of weekly variations in toxin levels, while longer time frame changes in toxin levels are mainly associated with external nutrient loading [21].

Although environmental monitoring agencies widely use the conventional methods, each of the methods suffers from specific limitations, including an inability to detect unculturable Cyanobacteria [22], microscopy requires extensive knowledge of phytoplankton structures and does not determine toxin production [23], the inability of PCR-based methods to distinguish between viable and non-viable cells [24], and potential cross-reactivity (false positive) using ELISA methods [25]. Additionally, conventional methods provide limited information regarding target-specific Cyanobacteria and cyanotoxins. However, cyanobacterial species/cyanotoxin levels differ from one environment to another [26], and limited information obtained from the conventional methods may not provide a comprehensive assessment for water quality monitoring.

Advancements in next-generation sequencing technologies have resulted in new analytical methodologies, such as metagenomics, that can provide a comprehensive view of microbial/cyanobacterial dynamics in recreational waters [27]. Amplicon sequencing/16S metabarcoding can provide only taxonomic information, while whole genome/shotgun sequencing can provide both taxonomic and functional changes in cyanobacterial communities [28]. Thus, implementing comprehensive information obtained from metagenomics with conventional methods can provide region-specific cyanobacterial trends, which can augment the water management/monitoring programs/strategies. However, the potential of metagenomics as an analytical technique still needs to be assessed and validated. In collaboration with the Ministry of the Environment, Conservation, and Parks (MECP), the current study evaluates the applicability and limitations of metagenomics and conventional ELISA and qPCR-based methods for cyanobacterial/algal bloom monitoring in the Great Lakes region. The questions addressed in this study are as follows: (1) Can metagenomics-based methods unambiguously identify Cyanobacteria species from complex recreational water samples? (2) Are there identifiable taxonomic and metabolic (functional genes) changes associated with bloom-enriched and non-bloom complex water samples? (3) Is there any correlation between MC/NOD concentrations and cyanobacterial 16S rRNA gene copies, as well as the correlation between *mcyE* and MC/NOD, and between *mcyE* and cyanobacterial 16S rRNA gene copies?

## 2. Results

A total of 108 lake water samples were collected and categorized into bloom and non-bloom samples using microscopy. All the samples were processed for 16S metabarcoding, and then a subset of samples (n = 24) showing ≥10% cyanobacterial abundance was used for shotgun DNA sequencing, microcystin ELISA, and cyanotoxin gene qPCR.

### 2.1. Quality Analyses

The data quality or variability in qualitative parameters can impact results from DNA sequencing, qPCR, and ELISA methods. Before the data analysis, we assessed each dataset for quality analysis. Supplementary Table S1 demonstrates the quality control analytics for cyanobacteria 16S and cyanotoxin qPCR analysis. Standard curve values used to determine gene copy counts in qPCR assays are also listed in Supplementary Table S1. The total cyanobacterial 16S rRNA assay, as well as *mcyE*/*ndaF*, *CyrA*, and *SxtA* assays, demonstrated high linearity between 10 and 100,000 gene copies/reaction, with coefficients of determination (R2) ranging between 0.9996 and 0.9999. Intercept and slope values for generated standard curves ranged between 38.5 and 39.14 and between −3.25 and −3.46, respectively. The calculated efficiencies of each standard curve ranged from 94.432% to 101.17%.

More than 90% of DNA sequencing reads passed the quality control criteria described in the methods section.

Supplementary Table S2 details the quality control analytics for ELISA analysis. The seven LFBs’ mean recovery was 117% ± 13%, demonstrating robust recovery. For the confirmation of the MRL (0.4 ng/mL), the upper prediction interval of results (PIR) limit was 115%, and the lower PIR limit was 54%. The five LRBs recovered less than 14% of the MRL to demonstrate an acceptable system background. For the analysis batch, the calibration curve had the coefficients a = 1.4, b = 0.73, c = 1.8, and d = 0.17, and a correlation coefficient (r2) of 0.99. Five standards had a coefficient of variation (CV) of less than 10%, and one standard had a CV of less than 12%. The four LRB wells recovered less than 5.8% of the MRL. The LFBs had recoveries between 112 and 140%. The low-CV had a recovery of 52%, and the QCS had a recovery of 85%. The LFSM/LFSMD had mean recoveries of 69–70%, CVs of 0.77–7.1%, and a relative difference of 1.0%. Lastly, the sample duplicates had CVs between 0.090 and 12%.

### 2.2. Taxonomic Composition Overview of Bloom and Non-Bloom Freshwater Lake Water Samples (Metabarcoding)

**Figure 1:**
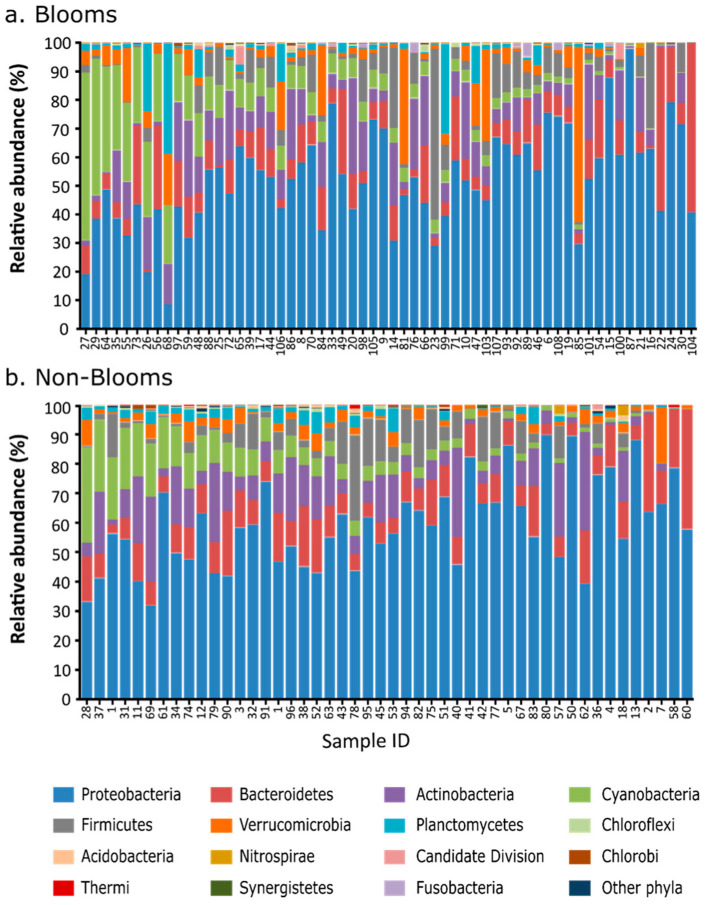
Taxonomic composition on phyla level for (**a**) bloom and (**b**) non-bloom samples using 16S metabarcoding.

All the samples (blooms and non-blooms) were first evaluated for overall taxonomic diversity using 16S metabarcoding (Figure 1). For non-bloom samples, Proteobacteria (32–85%) was the most dominant bacterial group, followed by Actinobacteria (4–35%), Bacteroidetes (3–30%), and Cyanobacteria (0.6–25%). Similar to non-bloom samples, Proteobacteria (19–75%) were the most abundant phyla in bloom samples, followed by Bacteroidetes (11–60%), Verrucomicrobia (5–60%), Cyanobacteria (2–60%), Actinobacteria (3–35%), and Planctomycetes (2–30%). Bloom samples also showed a higher prevalence of Planctomycetes and Verrucomicrobia than non-bloom samples.

### 2.3. Differential Taxonomic and Functional Composition of Bloom and Non-Bloom Freshwater Samples (Shotgun Metagenomics)

**Figure 2:**
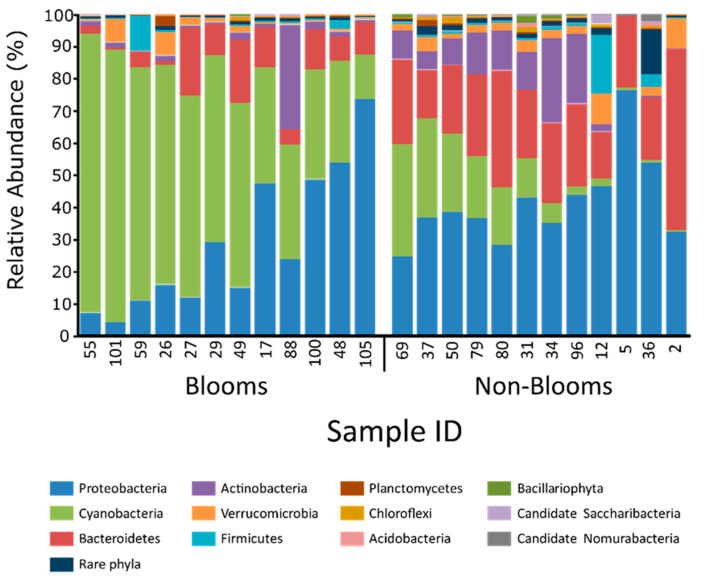
Taxonomic composition on phyla level for bloom and non-bloom samples using shotgun sequencing.

**Figure 3:**
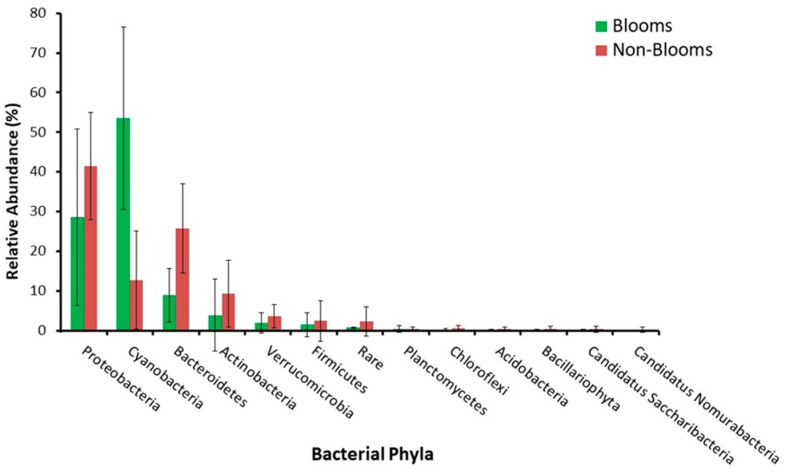
Differential abundance of bacterial phyla between bloom and non-bloom samples using shotgun sequencing.

Twenty-four samples (12 bloom and 12 non-bloom) were analyzed using shotgun metagenomics to characterize taxonomic and functional dynamics associated with bloom and non-bloom samples. At the phylum level (Figure 2), Proteobacteria (20–75%) was most dominant for all non-bloom samples, while Cyanobacteria (10–90%) was most abundant in seven (60%) bloom samples. Similarly, compared to non-bloom samples, cyanobacterial abundance was significantly higher (*p*-value = 1.2 × 10^−9^ in bloom samples (Figure 3 and Figure S1). Additionally, we observed an inversely proportional relationship between Bacteroidetes and Cyanobacteria for bloom samples. Cyanobacterial sequences were resolved to lower taxonomic levels to observe genus-level differences between bloom and non-bloom samples (Supplementary Figure S2). At the genus level, *Anabaena*, *Planktothrix*, *Microcystis*, and *Synechococcus* were abundant in both bloom and non-bloom samples (Supplementary Figure S2). Non-bloom samples were taxonomically (cyanobacterial) diverse, while bloom samples were dominated mainly by a single cyanobacterial genus (Supplementary Figure S2).

**Figure 4:**
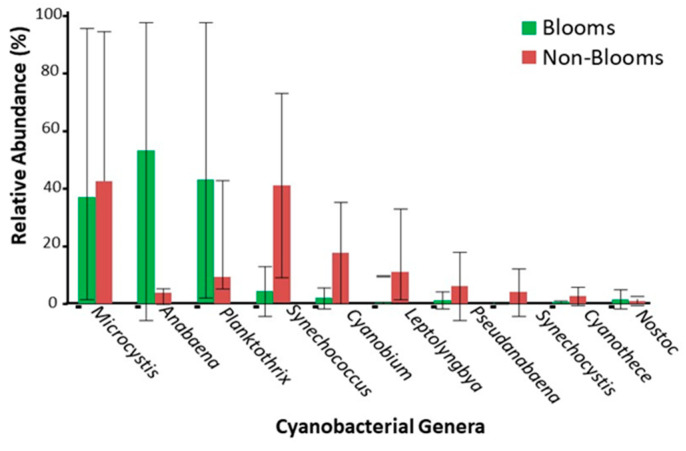
Differential abundance of bacterial genera between bloom and non-bloom samples using shotgun sequencing.

**Figure 5:**
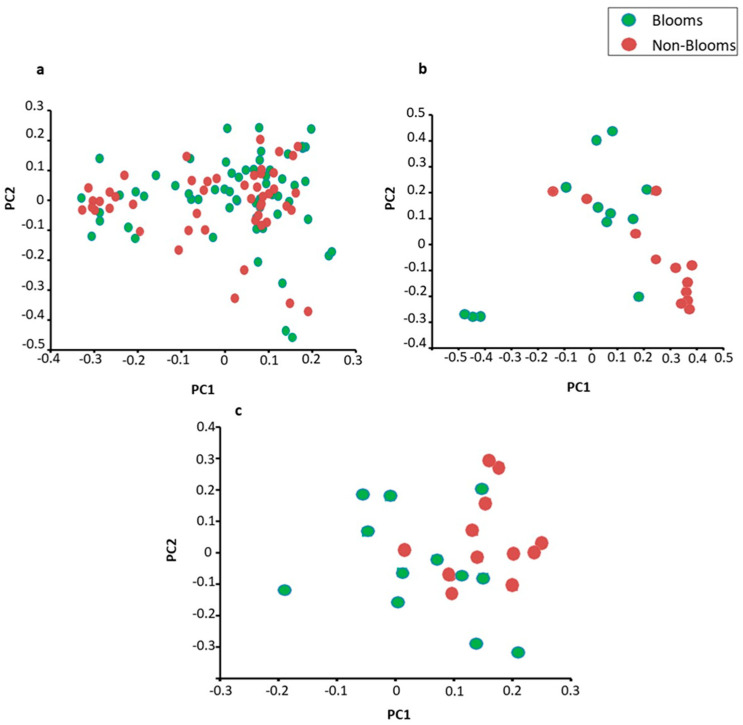
Beta diversity (PcoA) plots using weighted unifrac distance matrix on (**a**) order level (16S metabarcoding), (**b**) genus level (shotgun sequencing), and (**c**) functional (SEED subsystems level 2).

Overall differential abundance profiling identified that *Anabaena* (*p*-value = 3.3 × 10^16^) and *Planktothrix* (*p*-value = 9.1 × 10^−10^) were significantly more abundant in bloom samples than non-bloom samples (Figure 4 and Figure S3). The diversity analysis between the bloom and non-bloom samples on phylum and genus levels agglomerated most of the samples in close proximity to each other (Figure 5). However, diversity matrices using functional gene annotations showed differentiation between bloom and non-bloom samples.

Differential gene abundance profiling was performed to characterize specific functional differences between bloom and non-bloom samples (Supplementary Figure S4). Bloom samples demonstrated comparatively higher metabolic potential than non-bloom samples. Genes associated with cell division, cell wall, vitamins, co-factors and pigment synthesis, nucleic acid metabolism, cyanobacterial electron transport system, cyanobacterial heterocyst formation, and β-carboxysomes were differentially abundant in bloom samples compared to non-bloom samples. Additionally, genes associated with nitrogen and phosphorus metabolism were also more abundant in bloom samples (Supplementary Table S3).

### 2.4. Trends of Microcystin/Nodularin Levels and Cyanotoxin Gene Copies in Bloom and Non-bloom Recreation Water Samples

Table 1 shows the microcystin/nodularin concentrations, 16S rRNA gene copies, and *mcyE* gene copies of bloom and non-bloom samples. Three out of six (50%) bloom samples had MC/NOD levels (207, 73, and 3092 ng/mL, respectively) above the Health Canada recreational water guideline of 10 ng/mL, while two out of ten (20%) non-bloom samples (50, 69) had MC/NOD levels above the MRL, at 0.80 and 0.61 ng/mL, respectively. However, MC/NOD levels in non-bloom samples remained below the recreational water quality threshold.

Both bloom and non-bloom samples were positive for cyanobacterial 16S sequences (Table 1). Additionally, five out of six bloom samples (83%) showed the presence of microcystin gene copies, while only four out of 10 non-bloom samples (40%) showed the presence of microcystin gene copies. Furthermore, only one bloom sample demonstrated the presence of saxitoxin genes, and all the tested samples were negative for the cylindrospermopsin gene. The mean gene copies of total cyanobacterial 16S rRNA and *mcyE* per 5 ng of DNA showed no significant differences between bloom and non-bloom samples (*p*-values = 0.19 and 0.83, respectively). Interestingly, all the bloom samples were positive for microcystin-producing genera, but detectable microcystin levels were only observed for samples predominated by *Anabaena* and *Microcystis*. Correlation analysis was performed to observe the relationship between microcystin levels and cyanobacterial 16S/microcystin gene copies. Total MCs/NODs and *mcyE* gene copies were moderately (positively) correlated, with R = 0.52 and *p*-value = 0.038. Cyanobacterial 16S rRNA and *mcyE* gene copies were also moderately correlated, with R = 0.62 and *p*-value = 0.010. The correlation between total MCs/NODs and cyanobacterial 16S rRNA gene copies was non-significant (R = 0.44, *p* = 0.092).

## 3. Discussion

Cyanobacterial blooms are a global concern for the sustainability of water ecosystems, including the Great Lakes. Harmful algal blooms (HABs) can synthesize cyanotoxins that lead to the eutrophication of recreational waters [29,30]. Thus, continuous monitoring of water sources is necessary to avoid economic and health risks associated with the enrichment of Harmful algal blooms. Bloom development may not necessarily indicate toxigenicity, as not all cyanobacterial communities can produce cyanotoxins [7]. Additionally, cyanotoxin gene expression can be modulated by environmental factors, including nitrogen, phosphorus, and temperature [31], and conventional methods for cyanobacterial identification may not relate to cyanotoxin production or fully gauge the public health risks. Molecular techniques, including DNA sequencing, qualitative/quantitative PCR, and ELISA, can provide broad or targeted information that can circumvent the limitations associated with conventional methods. In this study, we are examining the potential of molecular technologies to augment traditional water monitoring strategies.

All the water samples (n = 108) were first analyzed using 16S metabarcoding to obtain a taxonomic overview of bloom-enriched and non-bloom samples. Verrucomicrobia and Planctomycetes sequences revealed a positive association with cyanobacterial abundance, which substantiates the studies that have identified these microbial taxa in eutrophic environments [32,33]. The association of Verrocomicrobia and Planctomycetes with cyanobacterial blooms can be due to a nutrient-rich environment, and both of these taxa have the potential to utilize polysaccharides synthesized by cyanobacterial communities [34,35]. Although 16S metabarcoding identified some taxonomic differences between the bloom and non-bloom samples, Proteobacteria sequences dominated most of the samples, and there was little difference in terms of cyanobacterial relative abundance. The inability of amplicon-based sequencing to differentiate between the bloom and non-bloom samples can be due to PCR bias that favors the over-amplification of dominant microbial taxa, including Proteobacteria [36,37]. Compared to 16S metabarcoding, shotgun metagenomics more efficiently characterized a greater abundance of cyanobacterial sequences in bloom-enriched samples, substantiating concerns that Cyanobacteria are the causative agents of many bloom formations in the Great Lakes region [38,39].

Additionally, shotgun sequencing was used to characterize the enrichment of specific cyanotoxin-producing genera in bloom samples, further validating the potential of using metagenomics sequencing-based methods to obtain more robust taxonomic profiles for bloom-specific response strategies. β-carboxysomes genes were differentially more abundant in bloom-enriched samples, and these genes are found predominantly in freshwater Cyanobacteria [40], which suggests Cyanobacteria to be the primary metabolically active component of microbial communities in the bloom-enriched water ecosystems. Although multiple metabolic pathways characteristic of cyanobacteria were differentially abundant in bloom-enriched samples, cyanotoxin genes were not detected in our datasets. Despite cyanobacterial DNA sequences being abundant, cyanotoxin genes may not represent a dominant proportion of total DNA [41], which hinders the detection of these genes without a higher depth of DNA sequencing [42]. For real-time detection purposes, qPCR or ELISA-based methods would better evaluate the toxigenic potential of bloom-enriched complex water samples. Currently, in Canada, there are no standard definitions for bloom designation at the federal or provincial level. While the initial assessments for bloom designation in this study were based on an initial microscopic assessment, more objective, quantitative criteria would be desirable that include comprehensive toxin identification and assessment. This is challenging because of the large number of microcystin congeners as well as the potential presence of other types of toxins (e.g., saxitoxin and anatoxin) that are not routinely monitored.

We observed only moderate positive correlations between cyanobacterial 16S rRNA and *mcyE* gene copies and between total MCs/NODs and *mcyE* gene copies, which is in agreement with previous studies from Canada [43], South Africa [44], and the USA [15,45], showing positive correlations between microcystins and *mcyE* gene copies. The correlation between total MCs/NODs and cyanobacterial 16S rRNA gene copies was non-significant (supported by shotgun sequencing), possibly due to bloom enrichment by non-toxic cyanobacterial communities [46]. Therefore, total cyanobacterial PCR markers may not be suitable for water quality assessment (testing cyanotoxin production) for freshwater ecosystems [41,47,48]. Additionally, samples with lower total cyanobacterial gene copies can still show high levels of cyanotoxins, which may be due to the proliferation of toxin-producing Cyanobacteria [49].

The filtration of LFBs using nylon syringe filters significantly reduced the MC/NOD recovery, likely due to the protein-binding properties of nylon filters [50]. USEPA method 546 for ELISA-based microcystin analysis recommends glass fiber filters [12], but other studies have reported success using regenerated cellulose and PES filters [10,45]. We did not observe any differences in microcystin recovery with the change in freezing temperature (−20 and −80 °C), which is in agreement with studies using different freezing temperatures for microcystin analysis [10,12,51]. Using crude DNA for qPCR resulted in variability of threshold cycle (Ct) values between the replicates, possibly due to PCR inhibition by cellular material [52,53]. However, crude DNA identified higher gene copies than purified DNA from the same samples, possibly because of DNA loss during purification cycles [54]. Therefore, DNA purification can reduce the PCR inhibition but may underrepresent the cyanotoxin gene copies. We did not observe cyanotoxin genes in metagenomics datasets (including bloom samples), which may be due to the requirement of higher sequencing depth for cyanotoxicity detection using DNA sequencing-based methods. Although conventional methods, including PCR and ELISA for cyanobacterial identification, can provide results within a few hours, they are limited in terms of information obtained and can be laborious for practical purposes when targeting a diverse cyanobacterial population. In comparison, next-generation DNA sequencing-based methodologies, including metagenomics, can provide a broader and robust overview of complex cyanobacterial communities, which can be difficult to differentiate visually or require multiple genetic markers for identification [55]. Additionally, there can be site/region-specific proliferation of particular cyanobacteria [56], which may not be identified using conventional molecular markers. However, DNA sequencing-based methods can provide a comprehensive taxonomic profile, which can be used for developing more specific and region-specific monitoring strategies. Additionally, bloom formation may not necessarily indicate the proliferation of toxin-producing cyanobacterial communities, thus, DNA-sequencing methods should be supplemented with real-time detection methods, including ELISA and qPCR, for more robust identification of cyanobacterial diversity and cyanotoxicity. This study provides a comprehensive comparative overview of molecular methods for Cyanobacteria/cyanotoxin detection in freshwater ecosystems and can improve conventional water monitoring strategies.

## 4. Conclusions

Our study concludes the following, that in freshwater ecosystems:

1. Non-bloom samples showed higher cyanobacterial taxonomic diversity compared to bloom samples, suggesting the dominance of specific Cyanobacteria genera during bloom formation.
2. *Anabaena* and *Planktothrix* were predominantly abundant in bloom samples than in non-bloom samples, possibly due to these species being common HAB bloom-forming algae in the Great Lakes region. Additionally, detectable microcystin levels were only observed for samples with a higher relative abundance of *Anabaena* and *Planktothrix*, suggesting their role in cyanotoxicity.
3. Compared to non-bloom samples, bloom samples showed significant cyanobacterial metabolic gene diversity, including nitrogen and phosphorus genes, which may represent a higher nutrient uptake and processing due to bloom enrichment.
4. Microcystin/nodularin levels positively correlated with *mcyE* gene copy numbers, indicating that microcystin gene copies can robustly estimate microcystin production in freshwater ecosystems.
5. Cyanobacterial 16S rRNA gene copy numbers showed a significant positive correlation with *mcyE* gene copies but not with microcystin/nodularin levels. Total cyanobacterial abundance may not indicate cyanotoxin production in freshwater ecosystems.

## 5. Materials and Methods

### 5.1. Study Design and Sample Collection

**Figure 6:**
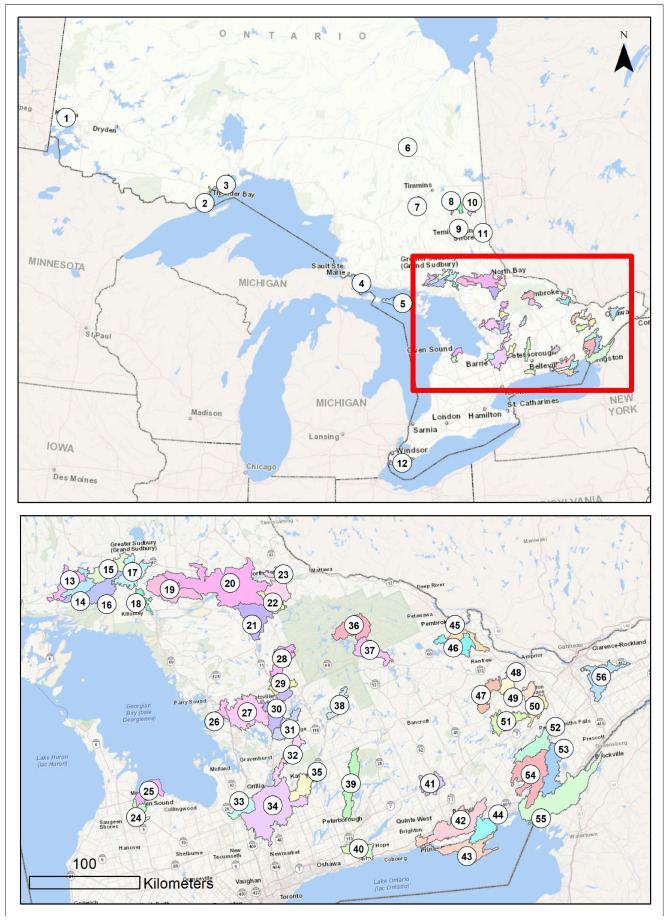
Sampling locations employed in this study. (Contains information licensed under the Open Government License—Ontario).

A total of 108 lake water samples were collected across the Great Lakes Region (Canada) by the MECP during the 2014 and 2015 sampling seasons (May to September). Figure 6 shows the locations of sampling sites for this study. Of these, 101 were collected from freshwater lakes, three from rivers, three from ponds, and one from a creek. The samples were categorized as bloom-enriched (n = 60) or non-blooms (n = 48) using light microscopy, and these same terminologies are employed throughout this manuscript. The samples were collected in 500 mL sterile polyethylene terephthalate (PET) bottles and stored at −80 °C. Samples were delivered to the McMaster lab in 2016 and stored at −80 °C until further use for DNA sequencing ELISA and qPCR testing.

### 5.2. DNA Extraction

Water samples were centrifuged at 10,000× *g* for 15 min. The supernatant was discarded, and the DNA was extracted from the cell pellet using the Norgen Soil Plus Extraction Kit (Norgen Biotek, Thorold, ON, Canada). Additionally, 10 μg/mL of lysozyme and 200 mM of β-mercaptoethanol were added to maximize the cyanobacterial cell lysis [57]. DNA was eluted in a final volume of 150 µL (TE Buffer) and stored at −80 °C.

### 5.3. DNA Sequencing Library Preparation

DNA extracted from all the samples (n = 108) was used for the Amplicon Sequencing (16S metabarcoding), while a smaller subset (n = 24, 12 bloom and 12 non-bloom) of samples was used for shotgun sequencing. For 16S metabarcoding library preparation, a 16S sequencing protocol (support.illumina.com/downloads/16s_metagenomic_sequencing_library_preparation.html; accessed on 1 June 2023) was followed. Briefly, the V3–V4 region (550 bp) of the bacterial 16S rRNA gene was amplified using DreamTaq Hotstart Mastermix (Thermo Scientific, Waltham, MA, USA). The PCR reaction mix was composed of 12.5 µL of master mix, 2.5 µL (1.0 µmole) of Forward Primer, 2.5 µL (1.0 µmole) of Reverse Primer, 5.0 µL of DNA, and 2.5 µL nuclease-free water. The amplicon PCR protocol included initial denaturation at 95 °C for 3 min, 35 cycles of 95 °C for 30 s, 55 °C for 30 s, and 72 °C for 30 s, followed by final extension at 72 °C for 5 min. Amplicon PCR products were purified using AmPure XP beads (Beckman Coulter Inc., Brea, CA, USA). Purified PCR products were subjected to index PCR using a Nextera XT index kit (Illumina Inc., Hayward, CA, USA). The second round of purification was performed using AmPure XP beads, and purified DNA libraries were pooled in equimolar concentrations after DNA quantification using QUBIT fluorometer (Thermo Scientific, MA, USA). All the PCR reactions were performed on a CFX-96 Touch PCR Detection System (BioRad, Santa Rosa, CA, USA). The shotgun sequencing library was prepared using Nextera XT DNA Sample Prep Kit (Illumina Inc., CA, USA). DNA sequencing was performed on MiSeq and HiSeq DNA sequencing platforms for 16S metabarcoding and shotgun sequencing at the Farncombe Sequencing facility (McMaster University, Hamilton, ON, Canada).

### 5.4. Metagenomics Data Analysis

Subsequently, 16S metabarcoding (Amplicon Sequencing) reads were processed using Quantitative Insights into Microbial Ecology (QIIME) using default parameters [58,59]. First, DNA sequence quality was estimated using FastQC [60], followed by adapter removal using Trimmomatic [61]. Sequencing reads were filtered to remove sequences with quality thresholds and lengths lower than 25 and 60, respectively. Quality-filtered reads were clustered into operational taxonomic units (OTUs) at 97% sequence similarity and assigned to their respective taxa using the RDP Classifier [62] against the Greengenes Database [63]. For shotgun sequencing, quality-filtered reads were aligned against the NCBI RefSeq database using the DIAMOND aligner [64], followed by taxonomic and functional annotation using Metagenome Analyzer (MEGAN) with an e-value cutoff of 1.0 × 10^−5^ [65,66]. All the statistical analysis was performed on R using the VEGAN [67] and DESeq2 [68] library packages.

### 5.5. Total Microcystins/Nodularins Determination by ELISA

ADDA-ELISA 96-well kits for microcystins/nodularins (MCs/NODs) were obtained from Gold Standard Diagnostics (Davis, CA, USA). Sample preparation was conducted according to of USEPA Method 546 [12]. Samples were lysed by three freeze–thaw cycles (freeze at −80 °C and thaw at 35 °C in a water bath) and filtered using 0.45 um PES membrane syringe filters (Cytiva, Marlborough, MA, USA). The immunosorbent assay was then conducted according to the manufacturer’s instructions (available at https://www.goldstandarddiagnostics.com/pub/media/productattachments/files/m/i/microcystins_nodularins-adda-elisa-user-guide-520011.pdf; accessed on 1 June 2023). The kit utilizes an indirect ELISA, with wells that are pre-coated with an analog of microcystins conjugated to a protein. In total, 50 µL of the standards, controls, and samples were first loaded into the wells in duplicates. This step was followed by adding 50 µL of the primary antibody solution. After 90 min of incubation, the wells were washed 3X with 1X Wash Buffer. Next, 100 µL of the secondary antibody–HRP conjugate solution was added to the wells, followed by 30 min incubation and three washes. A total of 100 µL of the color solution was then added to the wells, followed by 20 min of incubation away from sunlight and the addition of 50 µL of the stop solution. The absorbance of each well was determined at 450 nm with a BioTek Synergy H1 Multimode Microplate Reader (Agilent, Santa Clara, CA, USA) within 15 min of adding the stop solution. Samples that exceeded the maximum standard concentration were diluted with the sample diluent/LRB provided in the kit.

### 5.6. ELISA Quality Controls

Microcystin-LR (MC-LR) standards (10 ug/mL in methanol) were obtained from Gold Standard Diagnostics (Davis, CA, USA) [69]. The MC-LR standard was diluted to a concentration of 100 ng/mL and stored at −20 °C to be used for spiking the Laboratory Fortified Blank (LFB), Laboratory Fortified Sample Matrix (LFSM), and Laboratory Fortified Sample Matrix Duplicate (LFSMD). In the analysis batch, the following quality control elements were included: one quality control sample (QCS, 0.75 ng/mL), one LRB, two LFBs, one LFSM, and one LFSMD [12]. To confirm the assay response using primary calibration standards, a QCS provided along with the kit was assayed with each plate. The LRB was obtained from a reagent water source separate from the sample diluent supplied in the kit, and it was aliquoted into four wells throughout the plate. The LFB and LFSM/LFSMD were fortified with 1.0 ng/mL of MC-LR, and each was loaded into one well.

For the initial demonstration of capability (IDC), seven LFBs were fortified with 0.5 ug/mL of MC-LR to test precision and accuracy. The LFBs were then lysed, filtered, and assayed. Five LRBs were lysed, filtered, and assayed in the same analysis batch as the LFBs, including a low-range calibration verification (Low-CV) control to demonstrate an acceptable system background. To confirm the minimum reporting limit (MRL), seven LFBs were fortified at the proposed MRL concentration (0.40 ng/mL). The LFBs were then lysed, filtered, and assayed in an analysis batch containing one Low-CV, one QCS, and two LRBs. The threshold values for the IDC results are shown in Supplementary Table S2.

During MRL confirmation, the seven LFBs (0.40 ng/mL) were frozen at −80 °C. However, an additional three LFBs at the same MC-LR concentration were frozen at −20 °C to test if changes in freeze temperature led to changes in absorbance values/MC concentration. Welch’s *t*-test was performed subsequently to determine if there was a significant difference in the mean absorbances of the two sets of LFBs produced by the two freeze temperatures. Initial MRL confirmation tests were also filtered with 0.45 um nylon membrane syringe filters, which were later switched out for 0.45 um polyethersulfone (PES) membrane syringe filters (Cytiva, Marlborough, MA, USA). The absorbance values of the LFBs were compared using Welch’s *t*-test to determine if there was a significant difference in the mean recovery between the sets filtered using the two syringe filter membranes.

### 5.7. Cyanobacteria and Cyanotoxin Gene qPCR Analysis and Quality Control

Cyanobacterial and cyanotoxin gene copies were measured using qPCR analysis using CyanoDTec Total Cyanobacteria and Toxin Kit (Phytoxigene™, Akron, OH, USA) as per manufacturer’s instructions (available at https://static1.squarespace.com/static/531043b0e4b013842a3999f0/t/5d788d085bd75417004e0916/1568181527263/CyanoDTec+Procedure+Ver9.pdf; accessed on 1 June 2023). The cyanobacterial assay was used to measure 16S rRNA gene copy numbers, while cyanotoxin assays to measure microcystin/nodularin, saxitoxin, and cylindrospermopsin gene copy numbers. All qPCR reactions were run on the CFX96 Touch Real-Time PCR Detection System (BioRad Inc., CA, USA), and results were processed and analyzed using Bio-Rad CFX Maestro Version 4.1 software. Standard curves for the cyanobacterial and cyanotoxins were generated using CyanoDTec CyanoNAS (Phytoxigene™, OH, USA), followed by the generation of composite five-point standard curves for each assay with a range of 10–100,000 gene copies. Each qPCR reaction contained 20 µL of CyanoDTec master mix containing dNTPs, DNA polymerase, and primer–probe solution and 5 µL of extracted and purified DNA from samples. qPCR reactions were subject to initial denaturation at 95 °C for two minutes, followed by 40 cycles of 95 °C for 15 s, followed by 60 °C for 45 s. Gene copy numbers were calculated using the slope–intercept equation generated within the composite standard curve. A sample was confirmed to have 16S or toxin gene copies if the determined gene copy number was above the lower limit of the standard curve range and were only accepted as valid results if the internal amplification control threshold-cycle (Ct) value did not differ by more than 1.5 when compared to non-template controls.

qPCR reactions were run alongside non-template controls to monitor for inhibition or potential false positives. An internal amplification control (IAC) uses non-specific DNA and primers independent of cyanobacterial reactions as a gauge for whether samples are being inhibited by external sources (i.e., contaminants). As a challenge test, we tested crude and purified DNA from homogenous *Microcystis aeruginosa* culture before analyzing the samples for qPCR.

### 5.8. ELISA and qPCR Data Analysis

A four-parameter logistic calibration curve was generated using the absorbance (450 nm) of 6 MC-LR standards at concentrations of 0, 0.15, 0.40, 1.0, 2.0, and 5.0 ng/mL (provided by kit). Equation (1) was fitted to the absorbance values of the standards through regression analysis using the Solver add-in in Excel. This calibration curve was used to determine the MC/NOD concentration of the sample duplicates. The mean concentration of the two duplicates was then taken as the final concentration of MCs/NODs in each sample.

y = ((a − d))/(1 + (x/c)^b^) + d (1)

Equation (1): The four-parameter logistic calibration curve for ELISA analysis. As described in USEPA Method 546, y is the absorbance (450 nm), and x is the MC/NOD concentration. The coefficients, a, b, c, d, are calculated using logistic regression analysis.

Using the standard curve equation generated for the 16s rRNA and cyanotoxin gene copy assays, initial gene copy numbers are determined for each reaction using Equation (2).

g = 10^((−(w − m))/s)(2)

Equation (2): Conversion of observed Ct value to initial gene copy counts, where g is the initial gene copy count, w is the observed Ct value, and m and s are the y-intercept and slope of the generated standard curve, respectively.

The data for total MCs/NODs and cyanobacterial 16S rRNA/*mcyE* gene copies were tested for normality using Shapiro–Wilk’s (*p* < 0.05 for all three datasets), followed by Spearman correlation analysis (http://www.sthda.com/english/wiki/correlation-test-between-two-variables-in-r) accessed on 1 June 2023.

## Figures and Tables

**Table 1:** The microcystin/nodularin concentrations (ng/mL), total cyanobacterial 16S rRNA gene copies, *mcyE* gene copies, and *SxtA* gene copies of bloom and non-bloom samples. Only MC/NOD values above the MRL (0.40 ng/mL) were reported. Only gene copy number values higher than the lower limit of their standard curve range were reported. -—Not detected. *^a^*—Gene copies/5 ng of DNA.

Sample ID	Bloom Presence	Microcystins Nodularin (ng/mL)	16S rRNA *^a^*	*mcyE ^a^*	*SxtA ^a^*
26	Bloom	-	32,550	-	-
55		-	6013	11	-
59		210	10,039,974	534	86
100		73	23,499	15	-
101		-	6,430,707	15	-
105		3100	18,744	78	-
2	Non-bloom	-	102	-	-
5		-	3851	-	-
12		-	104,903	-	-
31		-	16,194	-	-
34		-	170	-	-
37		-	107,731	-	-
50		0.80	266,112	453	-
69		0.61	145,307	92	-
79		-	43,904	33	-
96		-	61,219	67	-

## Data Availability

The data presented in this study are available on request from the corresponding author due to sponsor privacy restrictions.

## Supplementary Materials

The following supporting information can be downloaded at: https://www.mdpi.com/article/10.3390/toxins16060263/s1, Figure S1: Differentially Abundant Phyla. The positive log2FoldChange indicates enrichment at bloom sites, and the negative log2FoldChange shows enrichment at non-bloom sites. The threshold-adjusted *p*-value for significance is 0.05; Figure S2: Relative Abundance of cyanobacterial genera for (a) Bloom and (b) Non-bloom samples using Shotgun Sequencing; Figure S3: Differentially Abundant Cyanobacterial Genera. The positive log2FoldChange indicates enrichment of taxa at bloom sites. The associated *p*-values for each cyanobacterial genera are indicated. The threshold-adjusted *p*-value for significance is 0.05; Figure S4: Differential Abundance of Functional Analysis. The SEED subsystems database was used at level 2 for the differential abundance of functions. Positive log2foldchange indicates differential abundance at bloom sites and negative log2foldchange at non-bloom sites. The threshold of significance was an adjusted *p*-value of 0.0001; Table S1: Quality control analytics for DNA Sequencing and qPCR analysis; Table S2: Quality control analytics for ELISA analysis, including control elements from the Initial Demonstration of Capability and the Analysis Batch; Table S3: Differential Abundance of Nitrogen and Phosphorus-associated Functions. The adjusted *p*-value cut-off is 0.05. Positive L2FC indicates enrichment at bloom sites.
