# Supplementary material for: Characterization of Taxonomic and Functional Dynamics Associated with Harmful Algal Bloom Formation in Recreational Water Ecosystems
Source: Toxins (Basel). 2024 Jun 7;16(6):263. doi: 10.3390/toxins16060263 (PMC11209277; doi:10.3390/toxins16060263)
Supplement: Supplementary file 1 [file toxins-16-00263-s001.zip › toxins-3001299-supplementary.pdf]

toxins

Supplementary Materials: Characterization of Taxonomic and Functional Dynamics Associated with Harmful Algal Bloom Formation in Recreational Water Ecosystems

Faizan Saleem, Rachelle Atrache, Jennifer L. Jiang, Kevin L. Tran, Enze Li, Athanasios Paschos, Thomas A. Edge and Herb E. Schellhorn

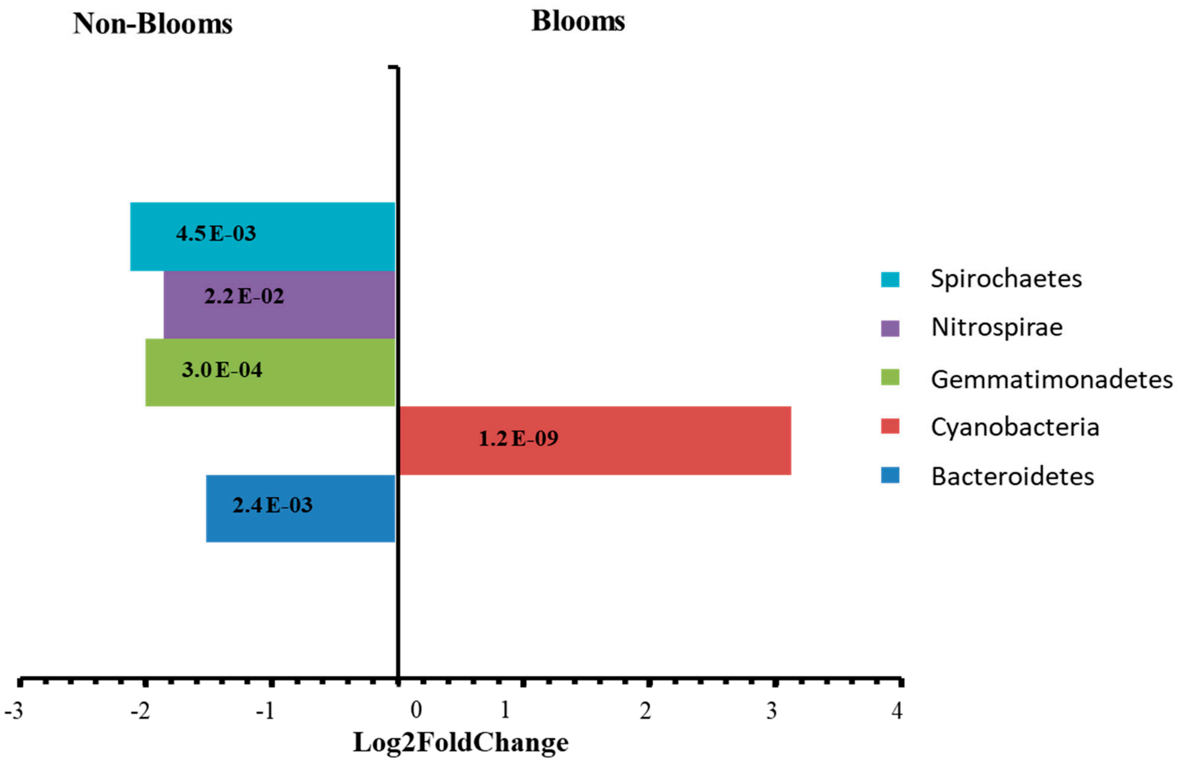

Figure S1. Differentially Abundant Phyla. The positive log2FoldChange indicates enrichment at bloom sites, and the negative log2FoldChange shows enrichment at non-bloom sites. The threshold-adjusted p-value for significance is 0.05.

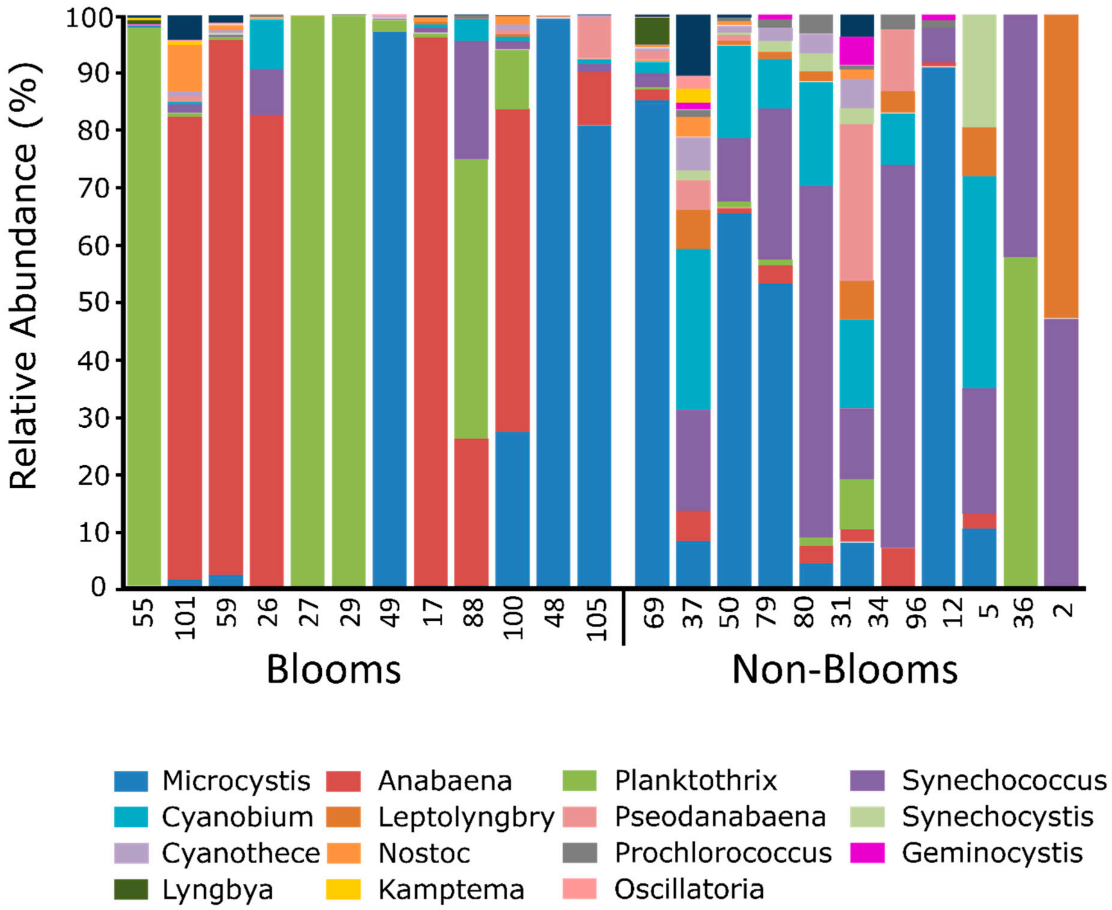

Figure S2. Relative Abundance of cyanobacterial genera for (a) Bloom and (b) Non-bloom samples using Shotgun Sequencing.

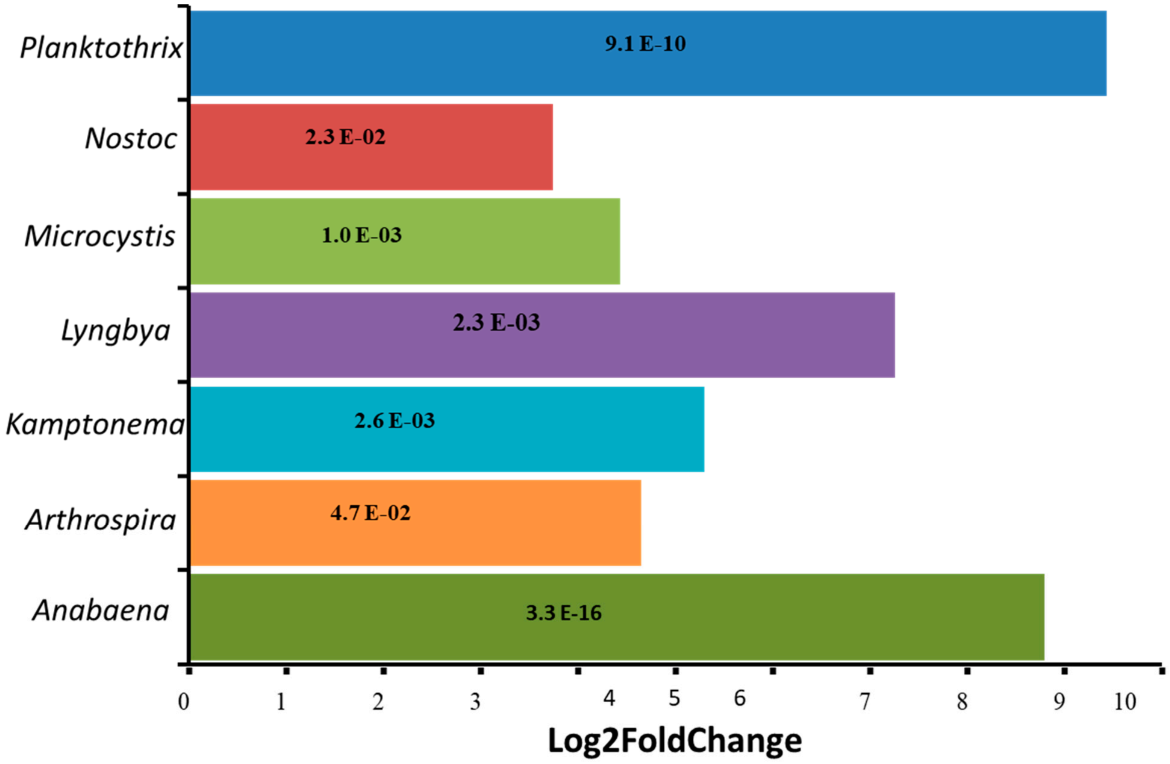

Figure S3. Differentially Abundant Cyanobacterial Genera. The positive log2FoldChange indicates enrichment of taxa at bloom sites. The associated p-values for each cyanobacterial genera are indicated. The threshold-adjusted p-value for significance is 0.05.

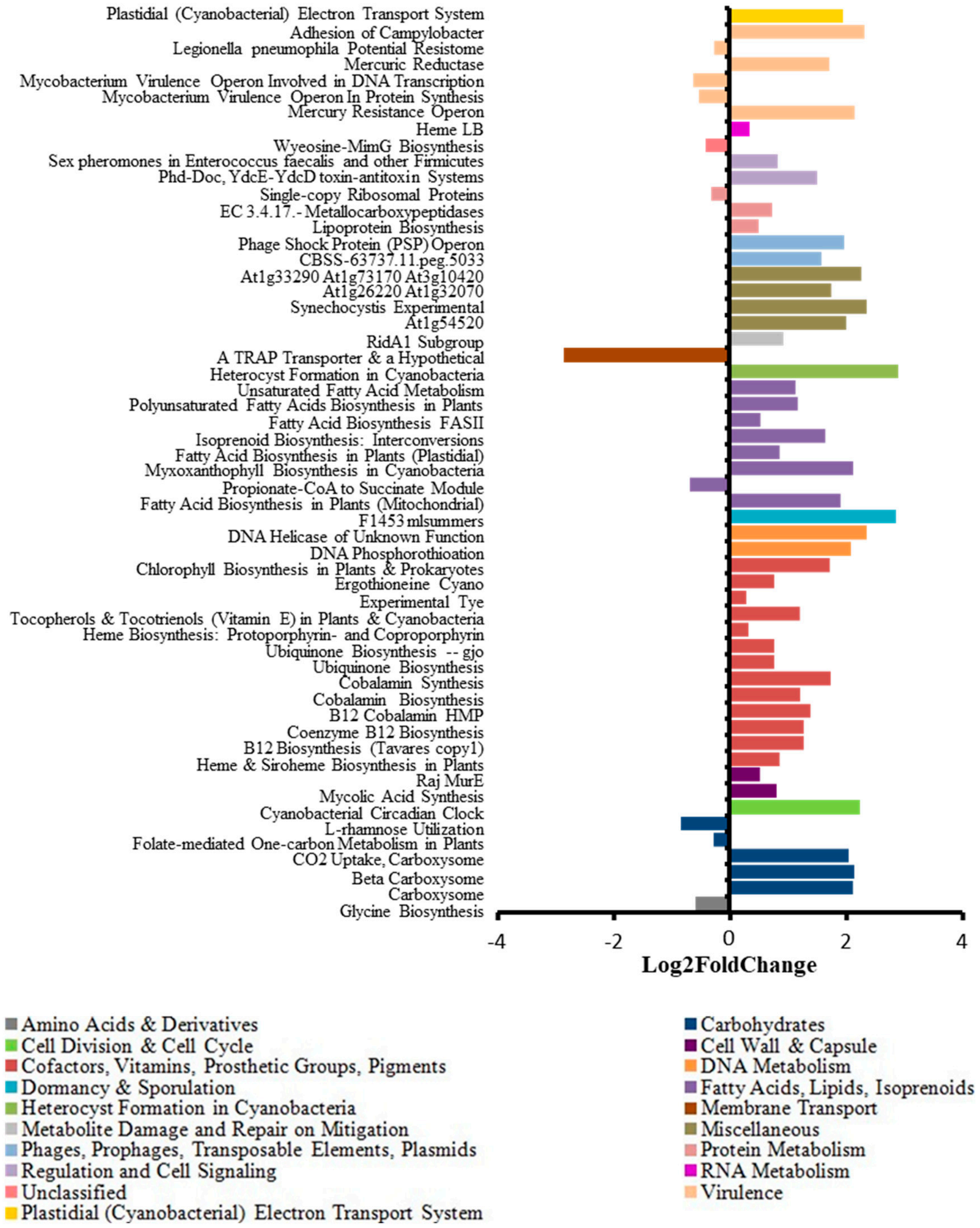

Figure S4. Differential Abundance of Functional Analysis. The SEED subsystems database was used at level 2 for the differential abundance of functions. Positive log2foldchange indicates differential abundance at bloom sites and negative log2foldchange at non-bloom sites. The threshold of significance was an adjusted p-value of 0.0001.

Table S1. Quality control analytics for DNA Sequencing and qPCR analysis.

**qPCR Standard Curve Values and Quality Parameters**		
**Assays**	**Quality Parameter**	**Value (Mean ± Standard Deviation)**
**Total Cyanobacteria 16s rRNA**	Coefficient of Determination (R^2)	0.999 ± 0.0003
	Slope	-3.46 ± 0.02
	Intercept	39.14 ± 0.08
	Efficiency (%)	94
**Microcystin/Nodularin (*mcyE*/*ndaF*)**	Coefficient of Determination (R^2)	0.999 ± 0.0003
	Slope	-3.38 ± 0.05
	Intercept	38.5 ± 0.3
	Efficiency (%)	97
**Cylindrospermospin (*CyrA*)**	Coefficient of Determination (R^2)	0.999 ± 0.0001
	Slope	-3.29 ± 0.07
	Intercept	38.9 ± 0.4
	Efficiency (%)	101.17
**Saxitoxin (*SxtA*)**	Coefficient of Determination (R^2)	0.999 ± 0.0003
	Slope	-3.33 ± 0.04
	Intercept	38.6 ± 0.3
	Efficiency (%)	99

Table S2. Quality control analytics for ELISA analysis, including control elements from the Initial Demonstration of Capability and the Analysis Batch.

**Control Element**	**Quality Parameter**	**Value**	**Threshold**
**Initial Demonstration of Capability**			
**LFBs to demonstrate precision and accuracy**	Mean Recovery	117%	≥70% and ≤130%
	Relative Standard Deviation	13%	≤15%
**LFBs to confirm the MRL**	Upper PIR Limit	115%	≤150%
	Lower PIR Limit	54%	≥50%
**LRBs to demonstrate acceptable system background**	Recovery (% of MRL)	<14%	≤50%
**Analysis Batch**			
**Calibration Curve**	Coefficients a, b, c, d (Equation 1)	a=1.4, b=0.73, c=1.8, d=0.17	-
	Square of the Correlation Coefficient (r^2)	0.99	≥0.98
**Standards**	CV	'<12%	≤15%
**LRBs**	Recovery (% of MRL)	<5.8%	≤50%
**LFBs**	Recovery	112-140%	≥60% and ≤140%
**Low-CV**	Recovery	52%	≥50% and ≤150%
**QCS**	Recovery	85%	≥70% and ≤130%
**LFSM/LFSMD**	Mean Recovery	69/70%	≥60% and ≤140%
	CV	0.77/7.1%	≤15%
	Relative Difference	1.0%	≤40%
**Sample Duplicates**	%CV	0.090-12%	≤15%

Table S3. Differential Abundance of Nitrogen and Phosphorus-associated Functions. The adjusted p-value cut-off is 0.05. Positive L2FC indicates enrichment at bloom sites.

**Function**	**baseMean**	**Log2FoldChange**	**lfcSE**	**stat**
Nitrogen Fixation	412.0183	0.927	0.492	1.883
Phosphorus Metabolism	10783.05	0.257	0.112	2.304
